# Comparative evaluation of intraosseous heat generation during bovine bone graft harvesting using two collector systems: a linear regression analysis

**DOI:** 10.1038/s41598-026-52740-z

**Published:** 2026-06-06

**Authors:** Ivan Calderon-Cortez, Sebastian Contreras, Lucia Quispe-Tasayco, Franco Mauricio, Fran Espinoza-Carhuancho, Ricardo Velasquez-Morales, Frank Mayta-Tovalino

**Affiliations:** 1 Periodontology Department, Faculty of Dentistry, Universidad Nacional Mayor de San Marcos, Lima, Peru; 2 EVIDENTIA Research Group, Universidad Nacional Mayor de San Marcos, Lima, Peru; 3 Faculty of Dentistry, Research, Innovation and Entrepreneurship Unit, Universidad Nacional Federico Villarreal, Lima, Peru; 4 Vicerrectorado de Investigación, Universidad San Ignacio de Loyola, Lima, Peru

**Keywords:** Autologous bone, In vitro, Graft harvesting, Heat generation, Biological techniques, Plant sciences

## Abstract

To compare heat generation within bone during the collection of autologous bone grafts using two different collection systems under controlled laboratory conditions. Forty D2-type bovine rib fragments were collected from an authorized slaughterhouse after the animals had been slaughtered for food. The samples were randomly divided into two groups: OSBC and Artifol. In each group, two types of drills (Ø4 mm and Ø5 mm) and two irrigation temperatures (room temperature and 10 °C) were tested. Perforation was controlled and temperature was measured with a Fluke TiS55 + thermal imaging camera. Statistical analysis included normality tests (Shapiro–Wilk), comparison of means (Student’s t-test), and multiple linear regression, all with a 95% confidence level and using Stata 17.0. The findings from the regression analysis indicated that using the Artifol System resulted in an increase in intra-osseous temperature by approximately + 0.92 °C (95% CI 0.30–1.55; *p* = 0.005). There was also a borderline increase in temperature produced with a Ø4 mm bur compared to a Ø5 mm bur (+ 0.32 °C; 95% CI 0.00–0.64; *p* = 0.048). When using cooled irrigation (10 °C), there were no longer any significant differences between the two systems and the OSBC system showed a slight decrease in temperature (− 0.18 °C; *p* = 0.301). In order to help establish this model further, the intercepts for both models showed baseline intraosseous temperatures of 13.40 °C and 14.82 °C, under ambient and cooled conditions respectively, providing further evidence of the superiority of irrigation as a means of thermal management. The type of collector system and the size of the bur will affect heat generation in bone, especially if the cold irrigation is discontinued. The continued application of irrigation at 10 °C has a strong effect on heat generation by the drill bur and results in the reduction of differences generated by devices and reinforcing its utility as a preventative technique in bone surgery.

## Background

Dental implants are considered surgically inserted attachments in the maxillary or mandibular bone to emulate the root of a missing tooth, which in turn can support a crown replacement such as a fixed partial denture^1^. Currently, this treatment modality is widely preferred for the prosthetic rehabilitation of a single tooth edentulous space due to its high success rates, even in the long term^2,3^, remaining functional in the mouth for up to 40 years^4^.

One of the requirements for the placement of dental implants is the presence of adequate bone volume, which guarantees the primary stability of the implant and its subsequent osseointegration^5^. However, after the extraction of a tooth, resorption of the surrounding alveolar bone inevitably occurs, which can lead to the formation of horizontal and/or vertical bone defects^6–8^.

To reverse bone loss after extraction and achieve dental implant success, bone grafts have been widely used as bone tissue substitutes^9^. Among the bone substitutes used in oral implantology are autografts (autologous bone), allografts (allogenic bone), xenografts (animal bone), and alloplastic grafts (synthetic material)^10^, the first of these being considered to date the gold standard for bone regeneration procedures^11,12^ due to its greater biocompatibility and because it has three important properties for the success of any type of graft: osteogenesis, osteoinduction, and osteoconduction^13^.

Over the years, various tools have been designed for the collection of autologous bones, some of the most studied being the bone chisel, bone scraper, bone filter, and trephine drill^14^. These drills are widely used in oral implantology to harvest bones from the patient (autologous bone) for the purpose of performing an autograft. However, this procedure involves the inherent generation of intraosseous heat, which is further increased by the continuous movement pattern required by these drills to obtain bone particles^15^.

The rise in intraosseous temperature has several repercussions on bone tissue, such as hyperemia, osteocyte degeneration, increased osteoclast activity, and finally osteonecrosis, which leads to dental implant failure due to the absence of osseointegration^16^. Despite the existence of several maximum temperature thresholds used as a reference for implant bed preparation, it has been determined that a temperature of 47° for 1 min is sufficient to cause bone tissue necrosis^17^.

Therefore, it is important for clinicians to take appropriate measures to minimize the potential impact that heat generation may have on the success of dental implant treatment. The aim of this study was to compare intraosseous heat generation during autologous bone harvesting using two harvesting systems by means of a linear regression model.

## Methods

### Ethics

This study did not involve ethical considerations, as it worked exclusively with bovine bone remains from an authorized slaughterhouse, where the animals were slaughtered for food purposes. The material used was collected after slaughter and without interfering with the production process, taking advantage of by-products that would otherwise have been discarded.

### Study design

This was an in vitro experimental study conducted under controlled laboratory conditions to compare how much heat is generated inside the bone when collecting bone grafts using two different collection systems, simulating a surgical environment as realistically as possible. The CRIS Guidelines (Checklist for Reporting in vitro Studies) were used for data reporting^18^.

### Sample size

To calculate the sample size, the classic formula for comparing means was applied, considering a statistical power of 80% (β = 0.8) and a confidence level of 95% (α = 0.05). The mean and standard deviation estimates were obtained previously through a pilot test, which allowed us to define a total sample of 40 specimens. These were distributed equally into two groups of 20, randomly assigned to the OSBC and Artifol collection systems (Table 1). In both groups, tests were performed with two irrigation temperatures (ambient and 10 °C) and 4 mm and 5 mm diameter burs were used to explore how these variables influenced heat generation during the procedure. All analysis was performed using Stata 17.0.

**Table 1:** Characteristics of bone collectors.

Bone collector	Manufacturer	Country	Diameters
OSBC	Dental Studio	South Korea	Drill Ø4, Ø5, Ø6, Ø7 mm
Artifol	Artifol Enterprises	Pakistan	Drill Ø4, Ø5, Ø6, Ø7, Ø8, Ø9, Ø10 mm

### Selection criteria

Fresh bovine rib fragments were selected from the Frigorifico La Colonial slaughterhouse in Callao, Lima, Peru (https://www.lacolonial.com.pe/historia.htm), days prior to the autologous bone collection procedure. Samples without visible damage and of adequate size and shape were included. Those with cracks, organic residues, or any alteration that could interfere with the measurements were discarded. Only D2-type bone was included.

### Allocation

The bone samples were distributed equally into two experimental groups, n = 20 for each, according to the collection system used: OSBC and Artifol. Each group was subdivided into four experimental conditions, combining two bur diameters (Ø4 mm and Ø5 mm) with two irrigation temperatures: room temperature (20–25 °C) and 10 °C. This assignment allowed for a systematic evaluation of the interaction between the drill diameter, the collection system, and the irrigation temperature.

### Preparation of bones

**Fig. 1:**
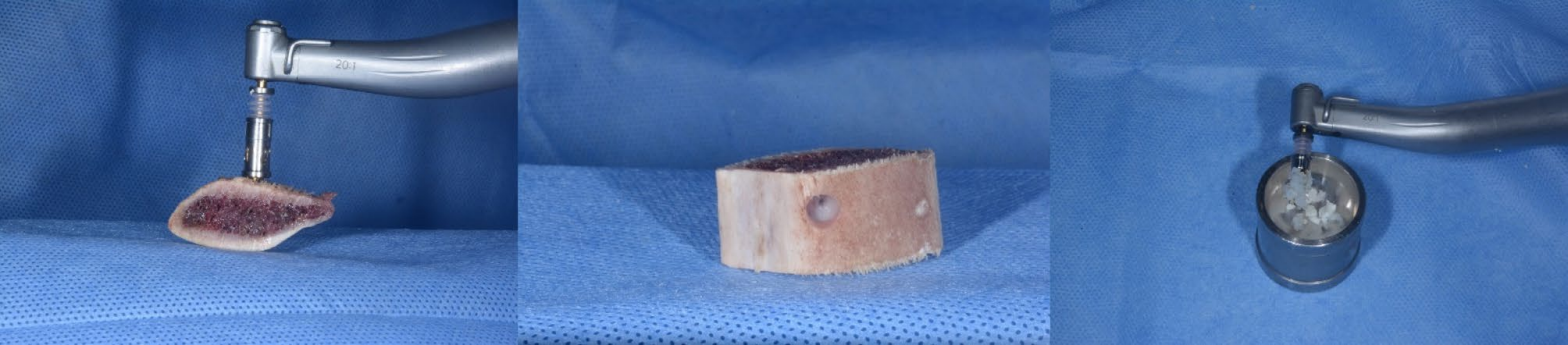
Obtaining autologous graft using bone harvester.

Segments of type D2 bovine ribs, characterized by moderate corticality and dense medullary bone, were collected from the Callao slaughterhouse (Lima, Peru). The bones were handled under strict hygienic conditions and transported to the laboratory in thermal containers, kept refrigerated at approximately 4 °C to preserve their thermal and structural properties. Transport and conditioning took no more than 6 h from the time of collection. Then, in the laboratory, the bones were carefully sectioned with a low-speed electric saw and constant cooling to avoid thermal artifacts. Uniformly sized blocks (approximately 5 cm) were obtained, which were fixed stably on a simulated surgical table for subsequent drilling (Fig. 1).

### Specimen preparation

**Fig. 2:**
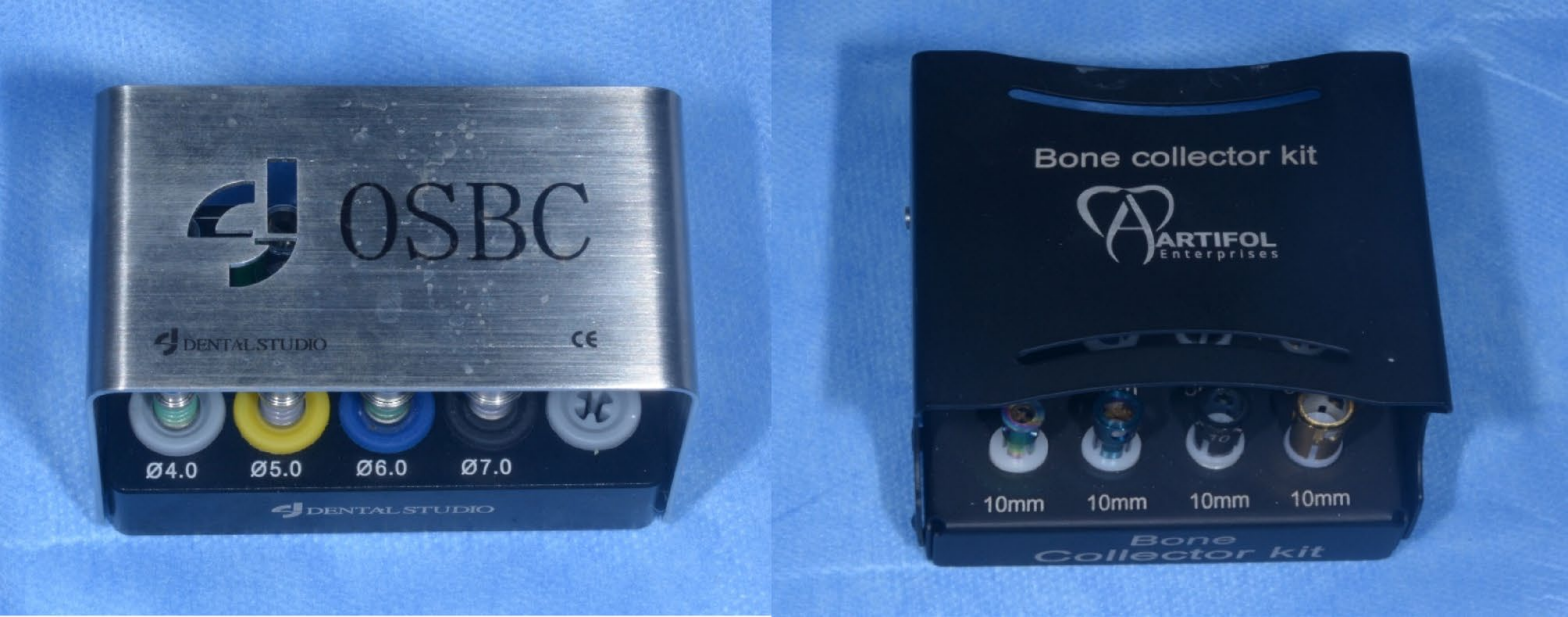
Bone harvester.

The specific storage conditions for the bone samples were stated; however, it was imperative that the samples were brought to an individual baseline temperature before drilling. Therefore, all the blocks were placed on cold packs at about 4 °C during transportation and allowed to acclimatize to the laboratory’s ambient temperature (22 to 24 °C) for a predetermined time before being drilled through. The graft was harvested using the two collection systems evaluated (OSBC and Artifol), following a standardized protocol that replicated clinical conditions. A micromotor with continuous external irrigation was used, employing cylindrical surgical steel drills in two sizes: Ø4 mm and Ø5 mm. Each perforation was performed at a perpendicular angle to the cortical surface, with controlled depth and duration (20 s per site), avoiding excessive pressure to simulate a conservative surgical technique. Each sample was used for a single perforation only, ensuring independence of measurements. All perforations were performed using a W&H implant motor, which provided stable rotational control and continuous external irrigation. The motor was coupled with a 20:1 reduction contra‑angle handpiece, ensuring precise torque transmission and consistent bur performance. Each drill was used a single time per specimen to ensure the independence of measurements and to avoid the confounding effects of drill wear on heat generation (Fig. 2).

### Thermographic evaluation

**Fig. 3:**
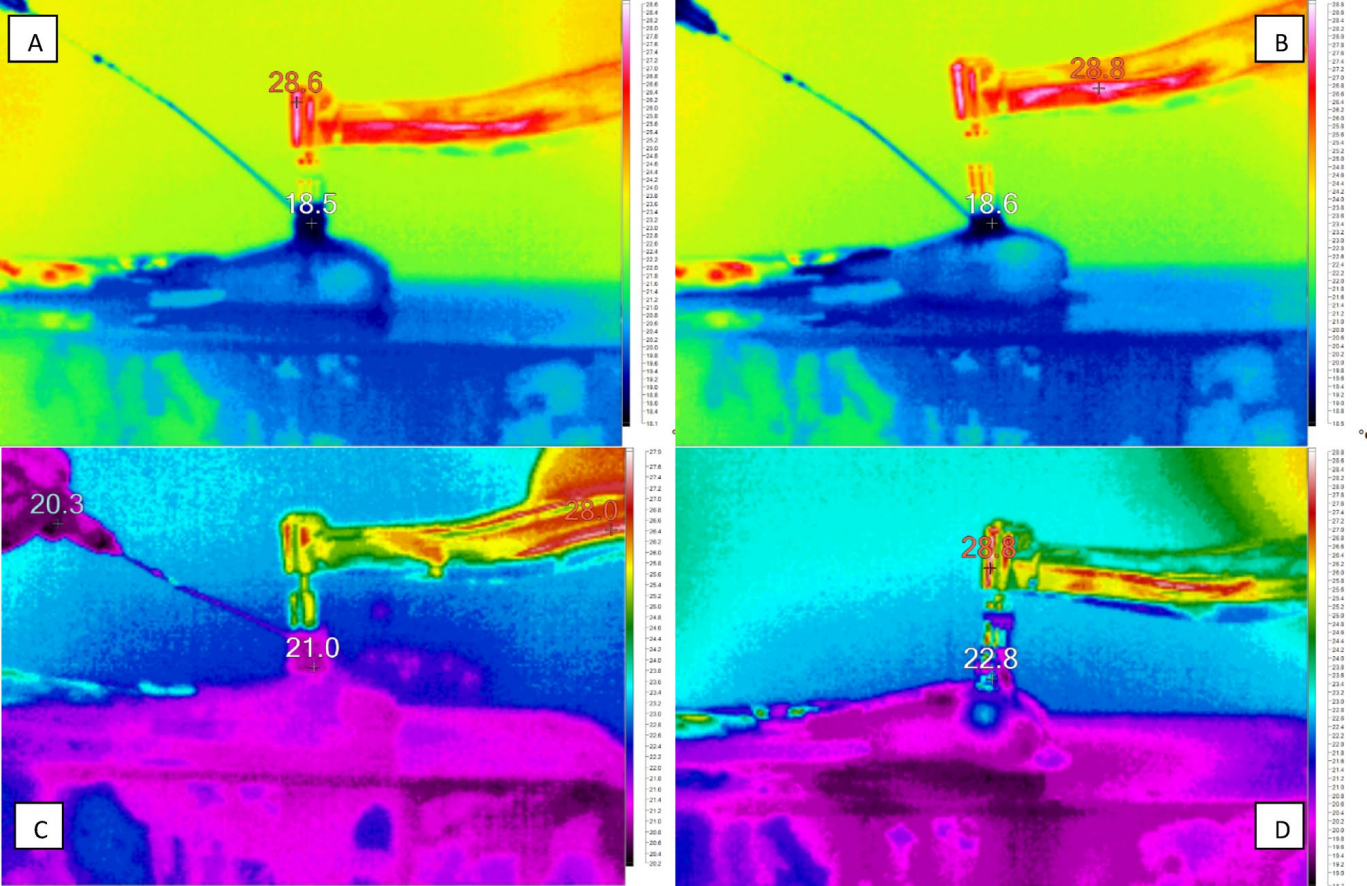
Infrared heat generated by bone harvesting systems when obtaining the autologous graft. (**A**, **B**) OSBC bone harvester system. (**C**, **D**) artifol bone harvester system.

The temperature generated during each drilling was measured using a Fluke TiS55 + infrared camera. This camera has an image resolution of 256 × 192 pixels (49,152 reading points), a spatial resolution of 1.91 mrad, and a field of view of 28° × 20°. The measurement distance was standardized to 0.5 m, and readings were taken immediately after drilling, recording the highest temperature point on the bone surface. All tests were performed under stable indoor light and environmental conditions (22–24 °C), and each block was allowed to cool completely before the next intervention (Fig. 3).

### Data analysis

The data were processed using Stata 17.0 software. In the first instance, the Shapiro–Wilk test was used to verify the normality of the variables. Afterwards, in order to mean-comparate the experimental groups, the Student’s t-test was used, always at the 95% confidence level. The homogeneity of the variances between groups was also verified prior to the comparative analyses in order to assure the accuracy of the results. A multiple linear regression model was then constructed to determine the effect of the collection system, irrigant temperature, and bur diameter on the temperature produced in the bone. The assumptions of the model (homoscedasticity, linearity, collinearity absent) were checked, and a *p* value < 0.05 used as the criterion for determining whether differences were statistically significant.

## Results

For each unit of change in the type of system (reference: Artifol), the average intraosseous temperature increased by 0.92 °C (95% CI 0.30 to 1.55), which was a statistically significant difference (*p* = 0.005). Likewise, the use of a Ø4 mm bur, instead of a Ø5 mm bur, was associated with an average increase of 0.32 °C in the final bone temperature (95% CI 0.00 to 0.64), with borderline significance (*p* = 0.048). The intercept of the model was 13.4 °C (95% CI 6.64 to 20.35; *p* = 0.000), representing the estimated base temperature under reference conditions (Table 2).

**Table 2:** Linear regression of final bone temperature irrigated with solution at room temperature.

Variables	Drill Ø5mm Irrigated at RT		
	Coefficient	*p*	β Crude
			CI 95%
Dental implant system			
Ref: Artifol	0.92	0.005	0.30 to 1.55
Drill Ø4mm irrigated at RT	0.32	0.048	0.00 to 0.64
_const	13.4	0.000	6.64 to 20.35

RT: Room temperature between 20–25 °C.

Under irrigation conditions at 10 °C, the use of the OSBC system (reference: Artifol) was associated with an average decrease of 0.18 °C in intraosseous temperature (95% CI − 0.55 to 0.17); however, this difference was not statistically significant (*p* = 0.301). On the other hand, the use of a Ø4 mm bur was associated with an average increase of 0.21 °C compared to the Ø5 mm bur (95% CI − 0.01 to 0.44), with a marginal p-value (*p* = 0.06), indicating a trend close to statistical significance. The intercept of the model was 14.82 °C (95% CI 10.8 to 18.80; *p* = 0.000), representing the estimated temperature under reference conditions (Table 3).

**Table 3:** Linear regression of the final bone temperature irrigated with solution cooled to 10 °C.

Variables	Drill Ø5mm Irrigated at 10 °C		
	Coefficient	p	β Crude
			CI 95%
Dental implant system			
Ref: Artifol	− 0.18	0.301	− 0.55 to 0.17
Drill Ø4mm irrigated at 10 °C	0.21	0.060	− 0.01 to 0.44
_const	14.82	0.000	10.8 to 18.80

## Discussion

The results of this study demonstrated that the application of a cooled irrigant (10 °C) during surgical preparations significantly reduces intraosseous heat generation, with no significant differences between each collection system regardless of the diameter used (Ø4 mm or Ø5 mm), which had a minor and insignificant impact on temperature elevation during the procedure.

On the other hand, when non-cooled irrigant or room temperature irrigant (20–25 °C) was used, there was a significant difference between the heat generated with the Artifol and OSBC collection systems, with the latter showing lower intraosseous temperature values, which were more noticeable with the Ø4 mm drill. This fact suggests that the diameter of the drill used is a factor influencing heat generation.

The failure rate of dental implants is strongly linked to the absence of osseointegration, which is influenced by various factors, one of the most important being thermal damage to the surrounding bone tissue. This increase in intraosseous temperature can be increased or decreased by variables related to the drill, the procedure, and the patient’s bone^19^. Therefore, the clinician must take certain precautions during the surgical procedure to avoid excessive temperature increases in the bone, prevent damage to it, and reduce the risk of osteonecrosis^20,21^.

The study by Omar et al.^22^, which compared the intraosseous heat generated during the preparation of the implant bed in bovine femurs using conventional drills, trephine drills, and alveolar expanders while irrigating externally with saline solution, determined that the lowest temperature rise occurred when using alveolar expanders (25.64 °C). while the highest temperature recorded was when trephine drills were used (28.26 °C). Therefore, to mitigate the impact of this type of drill, the authors of the study suggest the simultaneous use of external and internal irrigation. Unlike the current study, this study measured temperature using thermocouples, which were placed inside the bone samples.

Gehrke et al.^23^ compared the temperature variation in cortical bone during surgical preparation in artificial bone blocks using trephine burs and considering three irrigation methods (no irrigation, external irrigation, and combined irrigation). The results revealed that the highest temperature increase occurred in the group without irrigation, showing an average of 21.7 °C. On the other hand, the group that showed the lowest average intraosseous temperature was the one that used double irrigation (12.4 °C). These results are consistent with the recommendations of the study conducted by Omar et al., thus highlighting the use of simultaneous external and internal irrigation. This study measured temperature using thermocouples, and external irrigation was performed only at room temperature (25 °C).

The research conducted by Pintér et al.^24^ evaluated heat generation during osteotomy in pig ribs using trephine drills, taking into account the presence and absence of a surgical guide, as well as the application of external irrigation with saline solution. The group in which surgical guidance was used showed the maximum average temperature increase (7.9 °C) with respect to the initial temperature (20–23 °C). These results demonstrate the significant influence of the presence of surgical guidance during the preparation of the implant bed, a variable that greatly influences heat generation. This study, like the one now developed, used an infrared camera to measure temperature changes in bone tissue.

One of the strengths of this study includes the use of an infrared or thermographic camera (Fluke TiS55 + , Fluke, USA), which offers certain advantages such as: (1) no need for direct contact with the heat source, keeping the user out of danger; (2)the acquisition of two-dimensional images, which makes it possible to compare different areas of the measured object; (3) real-time temperature measurement, which allows for the evaluation of both stationary and moving objects, as well as the evaluation of rapid temperature variations; and 4) the non-invasive technique used by this device, which does not have a negative effect on the measured object. This last advantage makes it possible to use this device in vivo studies, since it is a non-contact technique that ensures a painless procedure for the animal or person being analyzed^25^.

Additionally, this study is the first to evaluate intraosseous heat using two different trephine systems and taking into account the temperature of the irrigant (cooled irrigant and room temperature irrigant), a variable that has been shown to have a significant influence on heat reduction during autologous bone collection for grafting, a fact that should be taken into account by clinicians from now on for this surgical procedure.

Our results on intraosseous temperature reduction through irrigation and collector systems resonate with the broader evidence that drilling parameters and bur characteristics play a decisive role in thermal behavior during osteotomy procedures. Soldatos et al. (2024)^26^ reported that in implant osteotomies, the counter‑clockwise (CCW) mode, particularly when combined with larger bur diameters (3.0 and 4.0 mm) and higher rotational speeds (800–1200 RPM), led to a significant rise in ΔT—exceeding 47 °C—and produced notable chatter after repeated use. In contrast, our study demonstrated that the OSBC system consistently achieved lower intraosseous temperatures than Artifol under room‑temperature irrigation, especially with Ø4 mm burs, and that cooling irrigation at 10 °C further minimized differences between systems. Taken together, these findings underscore the complex interplay between bur diameter, irrigation temperature, and system design in determining heat generation. While Soldatos et al. highlighted the risks associated with CCW drilling under specific conditions, our data suggests that optimized irrigation protocols and collector systems can serve as effective strategies to mitigate intraosseous temperature increases, even when larger burs are employed^26^.

This study underscores the importance of drill geometry and irrigation conditions in regulating intraosseous temperature during bone preparation. Soldatos et al. (2022)^27^ demonstrated that drill design and diameter exert a significant influence on ΔT in human tibiae, with tapered drills consistently generating more heat than straight drills, although none of the tested conditions surpassed the critical threshold of 47 °C. In our investigation, the OSBC system produced lower intraosseous temperatures than Artifol under room‑temperature irrigation, particularly with Ø4 mm burs, and cooling irrigation at 10 °C further reduced differences between systems. Taken together, these findings highlight that both drill design, as emphasized by Soldatos et al., and collector system configuration, as evidenced in our results, are key determinants of thermal behavior. While Soldatos et al. identified the thermal risks associated with tapered drills, our data suggests that appropriate irrigation protocols and optimized collector systems can mitigate these risks, thereby supporting safer conditions for bone graft harvesting and implant site preparation.

Our findings complement the evidence reported by Soldatos et al.^28^, who demonstrated that drill design, diameter, and rotational speed interact significantly to influence intraosseous temperature, although none of their tested conditions exceeded the critical threshold of 47 °C. In their study, Densah® burs produced a decrease in ΔT as diameter increased, whereas MIS® straight drills maintained or slightly reduced ΔT only at 1000 RPM. In contrast, our results showed that the OSBC system consistently achieved lower intraosseous temperatures than Artifol under room-temperature irrigation, with further reductions observed when cooling irrigation at 10 °C was applied. Taken together, these findings emphasize that beyond drill geometry and RPM, irrigation protocols and collector system design are decisive factors in thermal regulation. While Soldatos et al. highlighted the influence of drill design on heat generation, our data suggest that optimized irrigation strategies and collector systems can provide practical solutions to mitigate thermal risks, thereby supporting safer conditions for bone graft harvesting and implant site preparation.

An earlier study Jáni et al.^29^, indicated a low level of increase in temperature within the bone and the bone fragments collected during the harvesting process when the appropriate drilling parameters were employed using drilling devices. The average maximum intraosseous temperature in donor bones was observed to be lower than 10 °C, and the average maximum temperature of the bone fragments collected from the harvest process was lower than 5 °C, and therefore, are less than the critical/high-performance thresholds. Our results demonstrate that the intraosseous temperature created by the OSBC compared to Artifol was less at room temperature (compared to a cool temperature of 10 °C). The findings of our study further reinforce Jáni et al. findings that both the drilling characteristics of the instruments and the configuration of the collection devices are determining factors that will aid in lowering the risk of heat-related injuries that occur during the bone graft process. It is important to note that the findings of this study emphasize the clinical implications that are associated with the optimization of irrigation and the selection of devices used in the harvesting of bone grafts to minimize potential thermal injuries associated with the graft process.

According to our results, the systems used to collect bone (OSBC and Artifol) are able to dissipate temperature at room temperature, however when irrigating with water at a cooler temperature of 10 °C using a cooled irrigant, the cooler temperatures create strong thermal buffering, which has a much stronger influence than the configuration of the devices. At room temperature, the OSBC system had better temperature distribution than the Artifol system. Therefore, even though both designs have an influence on temperature regulation, due to the strong cooling effect created by the application of cooled irrigant during irrigating, the outcome is similar across the combined systems regardless of device configuration. There are slight differences in temperature generation due to differences in drill diameter, with the smaller diameter burs producing slightly higher temperature than larger diameter drills. This is due to the higher friction generated due to lower volume of cutting surface. This aspect of the study reinforces the conclusion that the configuration of the devices plays an important part in temperature control, however the temperature of the irrigant is the most important factor determining the thermal behavior of the device and the graft harvesting process.

Statistical analysis (regression analysis) verifies that differences between collector designs are statistically significantly different but should be interpreted as clinically relevant differences with caution. All temperature variations observed (less than 1 °C) were well below the critical injury temperature threshold of 47 °C sustained for one minute that has been widely identified in clinical studies as the temperature at which bone tissue sustains irreversible thermal injury. Therefore, in addition to the ability of the collector designs and drill bit diameter to affect and alter intraosseous temperature, they have a minor effect compared to the much larger effect of temperature in the irrigant when used as a cooling mechanism. Namely, using an irrigant at 10 °C negated the effects of the different collector systems, indicating that the temperature of the irrigant is the most significant factor influencing the thermal performance of tools when harvesting graft material. Smaller drill were also found to cause slightly higher localized temperatures around them when used under ambient conditions; however, these temperatures were not considered clinically significant or hazardous levels when comparing them to those achieved using larger diameter drills. Therefore, this study answers some questions related to how collector design, drill geometry, and irrigation interact with each other, as well as noting that although statistical significance exists, this does not necessarily mean there is an associated clinical risk.

This research had some limitations that should be considered. First, this in vitro study, carried out on bovine ribs (animal model), cannot reproduce 100% certain clinical conditions that normally occur in a person’s oral environment^22^. The reason for this is that the bone blocks used do not have blood flow, which may have some implication in the generation of intraosseous heat. Furthermore, these blocks cannot simulate the ambient temperature at which human alveolar bone is normally found when present in the mouth. Therefore, the intraosseous temperature increases generated in these samples may differ from those possibly obtained in human alveolar bone^30^.

## Conclusion

This in vitro study indicates that both the type of bone collector used and the measurements of the drill will affect the temperature of the bone throughout the duration of the harvesting process of an autologous graft. This is especially true if the method of irrigation used is at room temperature. Under this type of circumstance both the use of the OSBC and Artifol systems will have divergent effects on the temperature increase with OSBC being better at dissipating the temperature as compared to Artifol while using small burs. However, with a method of irrigation cooled to 10 °C difference between the two systems will be greatly diminished. This demonstrates the importance of the temperature of the irrigant in modulating the amount of heat generated in the bone. The results of the above tests using a bovine in vitro model can only be taken with caution and should not be directly related to the clinical applications of oral implantology and/or grafting of bone unless further studies (particularly human clinical trials) confirm the results will likely correlate to a human model.

## Data Availability

All data presented in this manuscript are available upon request from the corresponding author.

## Abbreviation

IS: In vitro study
